# ﻿First record of the genus *Cochleopsaltria* Pham & Constant (Hemiptera, Cicadidae, Cicadinae) from China, with a description of the second species

**DOI:** 10.3897/zookeys.1230.144099

**Published:** 2025-03-06

**Authors:** Cheng-Bin Wang, Zhi-Jian Liu

**Affiliations:** 1 Engineering Research Center for Forest and Grassland Disaster Prevention and Reduction, Mianyang Normal University, 166 Mianxing West Road, Mianyang 621000, Sichuan Province, China Mianyang Normal University Mianyang China; 2 School of Marxism, Fuzhou University, 2 Xueyuan Road, Fuzhou 350108, Fujian Province, China Fuzhou University Fuzhou China

**Keywords:** Cicada, Dundubiini, key, morphology, new genus record, new species, Oriental region, taxonomy

## Abstract

*Cochleopsaltria* Pham & Constant, 2017 (Hemiptera, Cicadidae, Cicadinae) is no longer monospecific: *C.huboliao***sp. nov.** from Fujian Province of China is described and illustrated. A key to the two species of *Cochleopsaltria* is presented.

## ﻿Introduction

The monotypic genus *Cochleopsaltria* (Hemiptera, Cicadidae, Cicadinae, Dundubiini, Dundubiina) was erected by Pham and Constant in 2017 for *C.duffelsi* Pham & Constant, 2017, based on a single male from northern Vietnam (Pham and Constant 2017). Since then, there has been no report on the discovery of this species or the genus.

In the present study, a new species of *Cochleopsaltria* is described from Huboliao National Nature Reserve, Fujian, China, representing a new national record for genus. Therefore, the number of the genera in the subtribe Dundubiina from China amounts to four: *Cochleopsaltria* Pham & Constant, 2017, *Dundubia* Amyot & Audinet-Serville, 1843, *Macrosemia* Kato, 1925 and *Platylomia* Stål, 1870 (Metcalf 1963; Chou et al. 1997; Sanborn 2013; Wang 2014). Illustrations of the diagnostic characters of the new species, a distribution map of the genus, and a key to the two species are provided.

## ﻿Material and methods

Four males and three females of the new species were collected from Huboliao National Nature Reserve of Fujian Province (China) on 25 October 2023. After fieldwork, the specimens were kept in a freezer (-20 °C). About half a year later, the specimens were relaxed and softened in water at room temperature for 12 h and then placed in distilled water for cleaning and dissection. To examine the male genitalia, the pygofer (containing the aedeagus), together with sternite VIII, were detached by fine-point tweezers and cleared with a trypsin solution at room temperature for 12 h. Then, they were placed in a 70% ethanol solution to remove the remaining trypsin. After examination, the body parts were mounted on a slide using Euparal mounting medium for future studies. Images were taken with a Canon MP-E 65 mm 1–5× macro lens on a Canon EOS 5DsR. Images of the same object at different focal planes were combined using Zerene Stacker 1.04 stacking software. Adobe Photoshop CS6 was used for post-processing. The description was carried out on dry specimens. Morphological terminology follows Moulds (2005, 2012) and higher taxonomy follows Marshall et al. (2018) and Simon et al. (2019). Measurement criteria in millimetres (mm) follow Wang and Liu (2022).

The type material of the new species is deposited in the following collections: **CLYQ**—Chonglinyequ Cultural Creativity Co., Ltd., Fuzhou, China; **MYNU**—Invertebrate Collection of Mianyang Normal University, Mianyang, China.

## ﻿Taxonomy

**Family Cicadidae Batsch, 1789**

**Subfamily Cicadinae Batsch, 1789**

**Tribe Dundubiini Atkinson, 1886**

**Subtribe Dundubiina Atkinson, 1886**

### ﻿Genus Cochleopsaltria

Taxon classificationAnimaliaHemipteraCicadidae

Pham & Constant, 2017

8014E0D4-AC8F-59C7-BBFE-53CB2FF19FD7

Cochleopsaltria Pham & Constant, 2017: 227 (description; 1 sp.); Marshall et al. 2018: 27, 39 (higher taxonomy; list).

#### Type species.

*Cochleopsaltriaduffelsi* Pham & Constant, 2017, by original designation and monotypy.

#### Note.

2 species; Oriental region.

#### Diagnosis.

The genus *Cochleopsaltria* Pham & Constant, 2017 can be separated from all other genera in the subtribe Dundubiina by the combination of the following characters: **Male.** Head not wider than pronotum while wider than mesonotum; rostrum at least reaching posterior coxae; pronotum shorter than mesonotum; pronotal collar with median length long, about 0.4 times as long as pronotal disc; forewings with infuscations; opercula spoon-shaped, broad and strongly convex in about apical 2/3, with apices broadly rounded, at least reaching posterior margin of sternite VI; abdomen almost as long as distance from head to cruciform elevation; pygofer upper lobes absent; uncus bifurcate with lobes stout, fused at base. **Female.** Abdominal tergite 9 with dorsal beak elongate, longer than anal styles; ovipositor sheath extremely elongate.

### ﻿ Cochleopsaltria duffelsi

Taxon classificationAnimaliaHemipteraCicadidae

Pham & Constant, 2017

9D1F9CA8-C2C2-5D91-B6BF-33D1A787C1B4

Cochleopsaltria duffelsi Pham & Constant, 2017: 227, figs 1–4 (description; illustrations).

#### Type locality.

“Hoa Binh 2, Quan Chu, Dai Tu, Thai Nguyen Province, 200–300 m”.

#### Distribution.

Vietnam.

### ﻿ Cochleopsaltria huboliao sp. nov.

Taxon classificationAnimaliaHemipteraCicadidae

14134308-549D-57E7-9A99-76E82BDB9859

https://zoobank.org/1AEF95BD-8D9B-41D4-B03F-FC0931EE64EA

**Figure 1.**
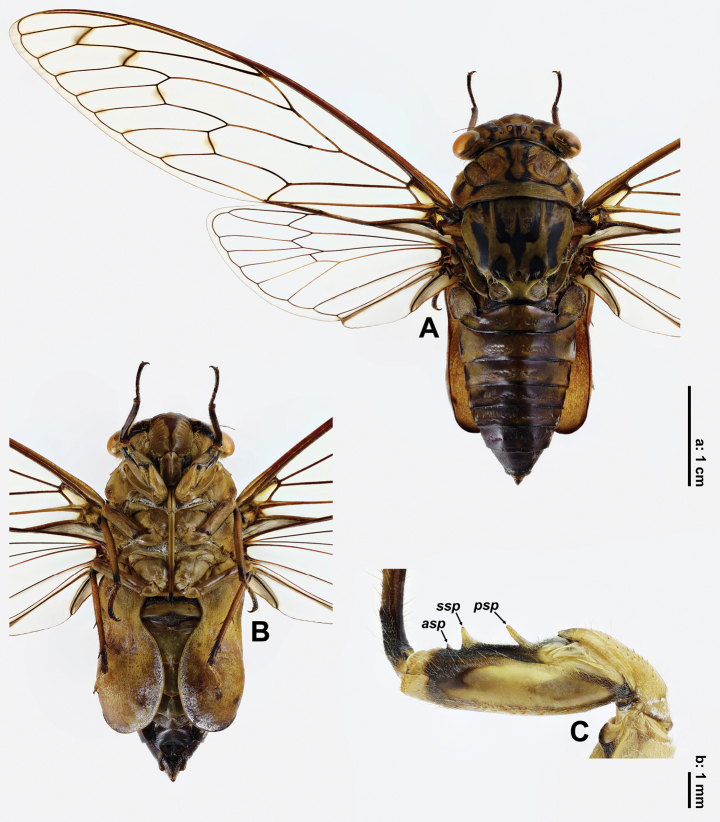
*Cochleopsaltriahuboliao* sp. nov., ♂, holotype **A** habitus, dorsal view **B** ditto, ventral view **C** right fore femur, lateral view. Abbreviations ***asp***: apical spine ***psp***: primary spine ***ssp***: secondary spine. Scale bar **a** for A, B and **b** for C.

**Figure 2.**
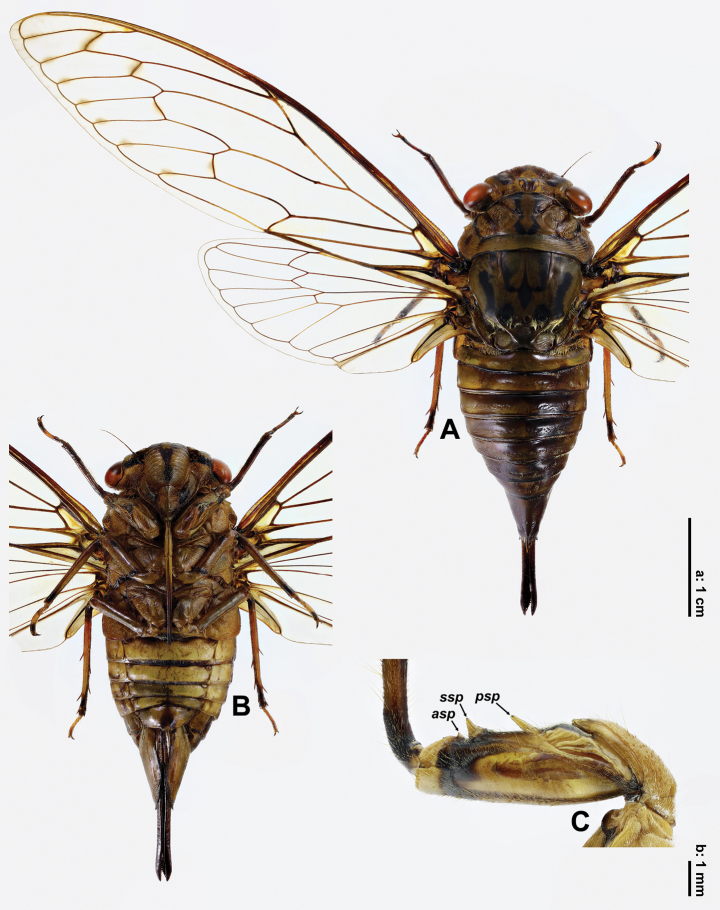
*Cochleopsaltriahuboliao* sp. nov., ♀, paratype **A** habitus, dorsal view **B** ditto, ventral view **C** right fore femur, lateral view. Abbreviations ***asp***: apical spine ***psp***: primary spine ***ssp***: secondary spine. Scale bar **a** for A, B and **b** for C.

**Figure 3.**
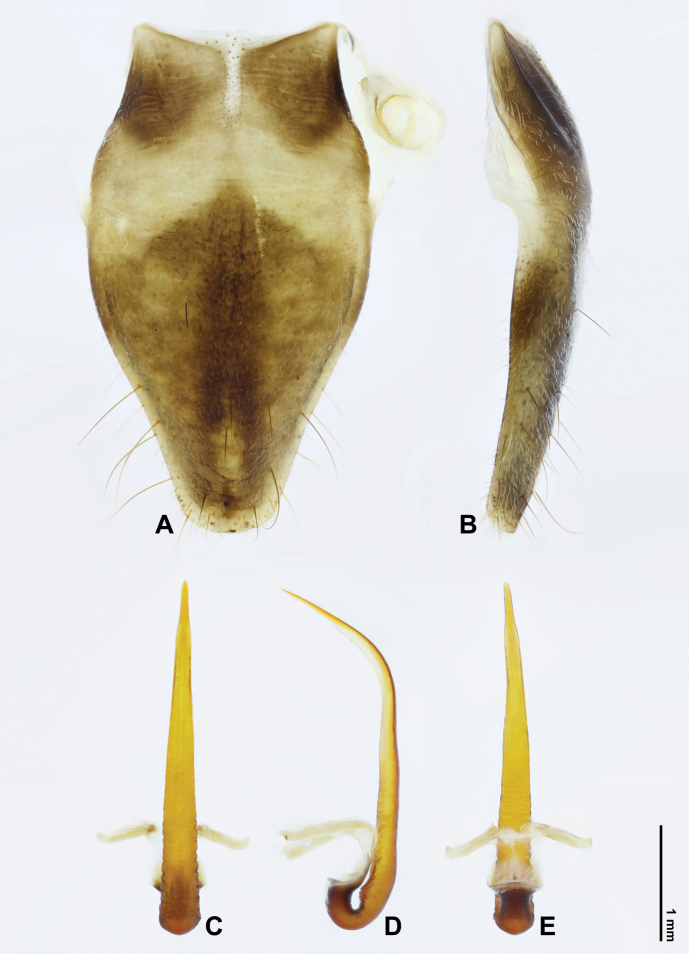
*Cochleopsaltriahuboliao* sp. nov., ♂, holotype **A** sternite VIII, ventral view **B** ditto, lateral view **C** aedeagus, dorsal view **D** ditto, lateral view **E** ditto, ventral view.

**Figure 4.**
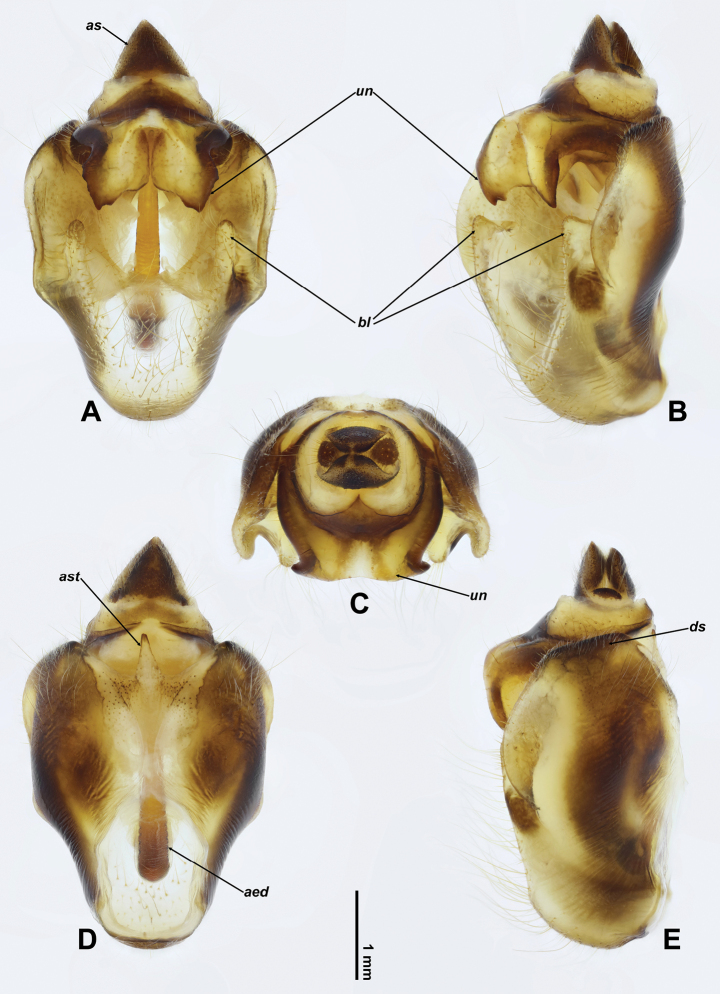
Pygofer of *Cochleopsaltriahuboliao* sp. nov., ♂ **A** ventral view **B** ventrolateral view **C** apical view **D** dorsal view **E** lateral view. Abbreviations ***aed***: aedeagus ***as***: anal styles ***ast***: apical stylus ***bl***: basal lobe ***ds***: distal shoulder ***un***: uncus.

**Figure 5.**
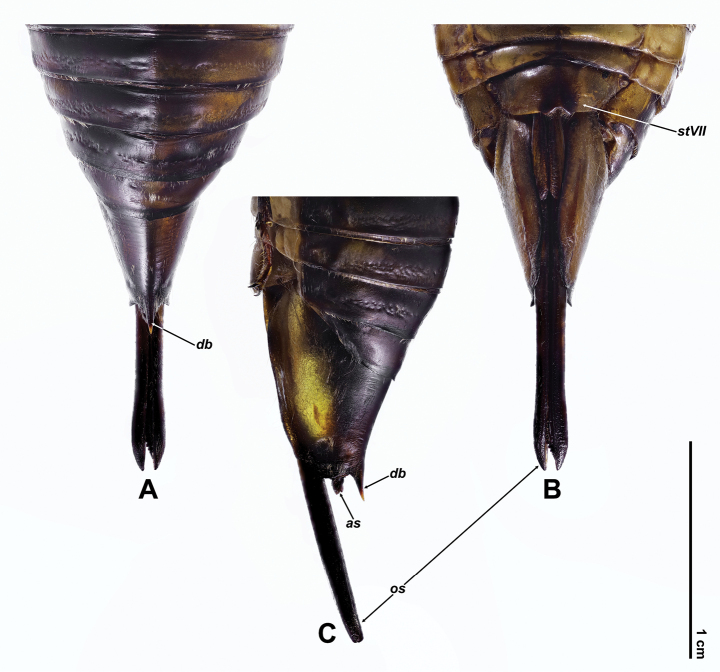
Female terminalia of *Cochleopsaltriahuboliao* sp. nov., paratype **A** dorsal view **B** ventral view **C** lateral view. Abbreviations ***as***: anal styles ***db***: dorsal beak ***stVII***: sternite VII ***os*** ovipositor sheath.

**Figure 6.**
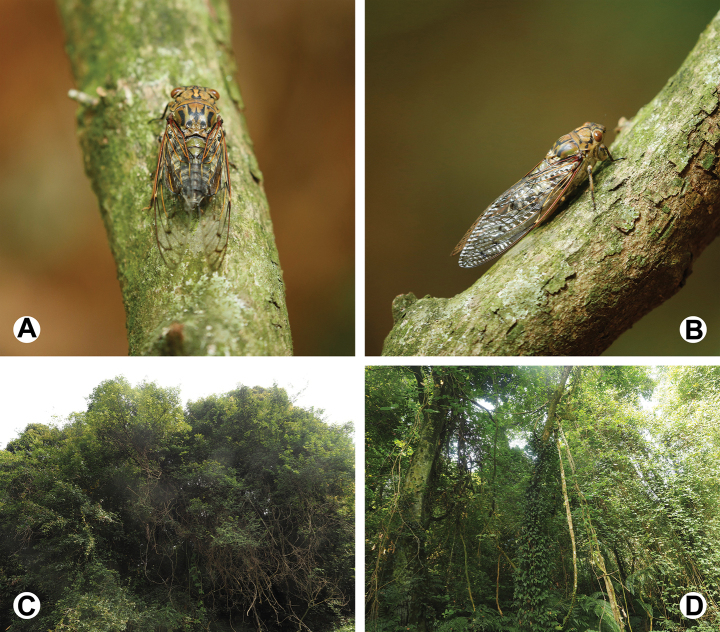
Field observations of *Cochleopsaltriahuboliao* sp. nov. at Huboliao National Nature Reserve (© Qun-Zhen Wu) **A** a living male perching on a branch, posterodorsal view **B** ditto, lateral view **C, D** habitat and host plant.

Figs 1A–C , 2A–C , 3A–E , 4A–E , 5A–C , 6A, B Chinese common name: 虎伯寮勺蝉

#### Type locality.

China, Fujian: Zhangzhou City, Huboliao National Nature Reserve [虎伯寮国家级自然保护区], Letu District [乐土片].

#### Type material.

4 ♂♂ 3 ♀♀. ***Holotype***: • ♂ (MYNU), **China, Fujian**: Zhangzhou City, Huboliao National Nature Reserve [虎伯寮国家级自然保护区], Letu District [乐土片], 25.X.2023, Liang Guo, Qun-Zhen Wu & Zu-Bin Chen leg. (MYNU). ***Paratypes***: • 3 ♂♂ 3 ♀♀ (1 ♀ in MYNU and 3 ♂♂ 2 ♀♀ in CLYQ), same data as holotype.

#### Etymology.

The specific epithet is from the Chinese name (in Pinyin) of the type locality “Huboliao”. The name is a noun in apposition.

#### Description.

**Male** (Figs 1A, B, 6A, B). ***Measurements*** (mm, *N* = 2, including smaller holotype). Body 37.3–39.2 long. Lengths of different body parts: head (2.6–2.9), pronotum (5.8–6.3), mesonotum (10.1–10.5), forewing (49.4–50.5), abdomen (18.8–19.5); width: head (13.0–13.2), pronotum (13.4–13.7), mesonotum (8.3–8.7), forewing (13.9–14.4), tergite 3 (12.0–12.3). Ratios of different body parts: (body length)/(head width) = 2.9; (pronotal length)/(head length) = 2.2; (mesonotal length excluding cruciform elevation)/(pronotal length) = 1.5; (abdominal length)/(head + pronotal + mesonotal length) = 1.0; (head width)/(pronotal width) = 1.0; (head width)/(mesonotal width) = 1.6; (tergite 3 width)/(mesonotal width) = 1.4; (forewing length)/(forewing width) = 3.6.

***Head*.** Ground colour ochraceous, with following black markings: longitudinal median fascia broad, bifurcate in posterior part, enclosing three ocelli, reaching frontoclypeal suture and posterior margin of head, without extended lateral parts; lateral fasciae broad except distinctly narrowed posterior part, reaching anterolateral and posterior margins of head; transverse fascia narrowly along posterior margin of head; paired small spots against posterior margin of head. Compound eyes ochraceous. Ocelli orangish. Distance between lateral ocellus and corresponding eye about 2.8 times as wide as distance between lateral ocelli. Antennae brown to fuscous. Postclypeus moderately swollen, mostly ochraceous, with paired brown oblique fasciae just anterior to frontoclypeal suture and a long “Y”-like brown median fascia ventrally, with 10–11 wide transverse grooves on each side. Anteclypeus ochraceous along median carina and brown laterally. Gena ochraceous, with oblique black fascia in anterior part. Lorum black in about posterior half and ochraceous in about anterior half. Rostrum ochraceous with blackish apical part, reaching middle of sternite II.

***Thorax*.** Pronotum ochraceous, slightly tinged with greenish on pronotal collar, with following black markings: submedian fasciae long, extending from anterior margin of pronotum to ambient fissure, broadened at both anterior and posterior ends; fascia along ambient fissure, broadening anterolaterally; two paired large elongate spots on pronotal collar, joining ambient fissure; no fasciae along paramedian and lateral fissures. Pronotal collar with median length long, about 0.4 times as long as pronotal disc, moderately ampliate posterolaterally; lateral margins with acute lateral teeth at about anterior 1/3, orientating laterally; posterolateral angles widely rounded; surface transversely grooved. Mesonotum greenish ochraceous, with following black markings: median fascia fusiform, broadened in middle part, strongly tapering anteriorly and reaching anterior 1/6 of mesonotum excluding cruciform elevation, moderately tapering posteriorly and not reaching anterior margin of cruciform elevation; submedian fasciae slender, along parapsidal sutures, moderately tapering anteriorly and broadening posteriorly, joining median fascia; accessory fasciae short, between submedian fasciae and accessory spots, not joining lateral fasciae posteriorly; lateral fasciae wide, gently curved, starting from anterior 2/7 of mesonotum excluding cruciform elevation, extending posteriorly just near anterior arms of cruciform elevation; accessory spots small, lateral to accessory fasciae; posterior spots large, occupying scutal depressions. Cruciform elevation bright ochraceous, with paired black markings on anterior arms. Wing groove ochraceous. Ventral side ochraceous, basisternum 2 with paired oblique black rhombic markings, basisternum 3 with paired small black spots, surface densely with short setae.

***Legs*.** Bicoloured, ochraceous to brown with blackish markings. Profemur (Fig. 1C) with three spines: primary spine slender, digitiform, obliquely inserted, with apex rounded; secondary spine subtrianglular, with apex rounded; apical spine rather small, subtrianglular, with apex rounded. Meracanthi ochraceous, slender and slightly curving medially.

***Wings*.** Hyaline. Forewing with eight apical cells; ulnar cell 3 about twice as long as apical cell 5; RA_2_ vein with distal portion about 1.9 times as long as proximal portion; venation color mixed with ochraceous, brown and fuscous; infuscations present on r, r-m, m and m-cu crossveins, and paler on apices of longitudinal veins of apical cells; nodal line absent; basal cell greyish ochraceous; basal membrane greyish ochraceous. Hindwing with six apical cells; venation color mixed with ochraceous, brown and fuscous, 3A blackish; jugum and longitudinal margins of vannus greyish ochraceous.

***Operculum*.** Mostly ochraceous, with basal part of lateral margin blackish; spoon-shaped, constricted around basal 2/7, broad and strongly convex in about apical 2/3; apex broadly rounded, extending beyond posterior margin of sternite VI; separated from each other about 1/5 width of one of them; lateral margin slightly bisinuate while medial margin strongly so.

***Abdomen*.** Obconical, in dorsal view generally brownish in basal part and fuscous apically. Tergite 1 fuscous to blackish; tergites 2–8 with posterior margins narrowly blackish; tergites 3–6, each with one fuscous spot at lateral side. Timbal cover oval, ochraceous with narrow brown margin, covered with greyish hairs, especially in lateral part, completely concealing timbal in dorsal view. Sternites III–VI mostly ochraceous to brown; sternite VII fuscous except ochraceous in posterior part, subhexagonal, inconspicuously emarginate at posterior margin, with longitudinal median groove in posterior part; sternite VIII (Fig. 3A) ochraceous with brown median fascia and paired anterior markings, drop-like, rounded at posterior margin, anterolateral apodemes subtriangularly developed.

***Genitalia*.** Pygofer suboval, more or less narrowing anteriorly in ventral and dorsal views (Fig. 4A, D); anal styles relatively large, moderately sclerotised, densely covered with short setae (Fig. 4A–E); apical stylus relatively large, slender, lightly sclerotised, digitiform (Fig. 4D); basal lobes in ventral view elongate (Fig. 4A), in ventrolateral view subtriangular in apical parts (Fig. 4B); upper lobes absent; distal shoulders obliquely truncated at apices in lateral view (Fig. 4E). Uncus bifurcate; lobes stout, fused at base, rather narrowly separated from each other medially, in ventral view angulate at apices, crenulate at lateral margin and bisinuate at medial margin (Fig. 4A), in ventrolateral view further one bidentate at apices (Fig. 4B). Aedeagus thin and slender, gradually narrowing towards apex, without processes (Fig. 3C, E); in lateral view, strongly turning ventrally in apical 1/3 and almost straight in basal 2/3 (Fig. 3D).

**Female** (Fig. 2A, B). *Measurements* (mm, *N* = 2). Body 42.1–45.8 long. Length of different body parts: head (1.9–2.7), pronotum (4.9–5.9), mesonotum (9.1–10.0), forewing (50.8–52.6), abdomen (26.2–27.2); width: head (12.6–13.1), pronotum (13.5–13.9), mesonotum (9.5–10.1), forewing (14.2–14.7), tergite 3 (13.1–13.5). Ratios of different body parts: (body length)/(head width) = 3.5; (pronotal length)/(head length) = 2.1; (mesonotal length excluding cruciform elevation)/(pronotal length) = 1.4; (abdominal length)/(head + pronotal + mesonotal length) = 1.5; (head width)/(pronotal width) = 1.0; (head width)/(mesonotal width) = 1.3; (tergite 3 width)/(mesonotal width) = 1.3; (forewing length)/(forewing width) = 3.6.

Rostrum extending beyond posterior margin of abdominal sternite II; profemur (Fig. 2C) similar to that of male; abdomen subconical, gradually converging apically; operculum short, rounded at posterior margin, extending slightly beyond posterior margin of abdominal sternite II and separated from each other by about 1.7 times width of one of them; abdominal sternite VII (Fig. 5B) subroundly incised at middle of posterior margin, with paired protuberances flanked incision; abdominal tergite 9 with dorsal beak (Fig. 5A, C) elongate, roundly sharp, longer than anal styles; ovipositor sheath (Fig. 5A–C) blackish, extremely elongate.

#### Variation.

All male or female types without evident variation.

#### Field observations.

A living male of the new species is shown in Fig. 6A, B and its habitat in Huboliao National Nature Reserve is shown in Fig. 6C, D. According to the collectors’ recollection, during the collecting trip in late October, this cicada was abundant in the reserve, but the individuals were difficult to capture. They were in the canopy and not attracted to light traps at night, so the collectors had to climb up the trees to capture them.

#### Distribution.

**Figure 7.**
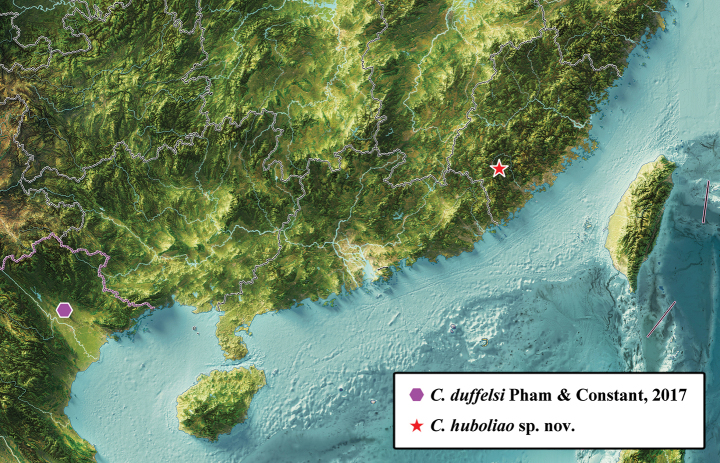
Distribution map of *Cochleopsaltria* species.

China (Fujian) (Fig. 7).

#### Differential diagnosis.

The new species well resembles its only congener *C.duffelsi* from Vietnam in general appearance, but it is not difficult to distinguish them using the following key.

Additionally, although many species in Cicadinae exhibit morphological variations in markings and operculum length, they still have certain reference characteristics:

In *C.duffelsi*, pronotal collar with three paired large spots (Pham and Constant 2017: figs 2A; 3A); mesonotum with median fascia moderately tapering anteriorly and weakly so posteriorly, lateral fasciae relatively slender, accessory spots absent (Pham and Constant 2017: figs 2A; 3A); opercula reaching posterior margin of sternite VI (Pham and Constant 2017: figs 2B; 3B). In *C.huboliao* sp. nov., pronotal collar with two paired large spots (Fig. 1A); mesonotum with median fascia strongly tapering anteriorly and moderately so posteriorly, lateral fasciae relatively wide, small accessory spots lateral to accessory fasciae (Fig. 1A); opercula extending beyond posterior margin of sternite VI (Fig. 1B).

### ﻿Key to males of *Cochleopsaltria* Pham & Constant, 2017

**Table:** 

1	Pygofer basal lobes in ventrolateral view rounded in apical parts (Pham and Constant 2017: fig. 4B); uncal lobes in ventral view rounded at apices, simply arcuate at both lateral and medial margins (Pham and Constant 2017: fig. 4C), in ventrolateral view further one rounded at apices (Pham and Constant 2017: fig. 4B)	***C.duffelsi* Pham & Constant, 2017**
–	Pygofer basal lobes in ventrolateral view subtriangular in apical parts (Fig. 4B); uncal lobes in ventral view angulate at apices, crenulate at lateral margin and bisinuate at medial margin (Fig. 4A), in ventrolateral view further one bidentate at apices (Fig. 4B)	***C.huboliao* sp. nov.**

## Supplementary Material

XML Treatment for Cochleopsaltria

XML Treatment for Cochleopsaltria duffelsi

XML Treatment for Cochleopsaltria huboliao

## ﻿Additional information

### Author ORCIDs

Cheng-Bin Wang https://orcid.org/0000-0002-7913-8779

Zhi-Jian Liu https://orcid.org/0009-0000-2129-1861

### Data availability

All of the data that support the findings of this study are available in the main text.
